# P2Y12 regulates microglia activation and excitatory synaptic transmission in spinal lamina II neurons during neuropathic pain in rodents

**DOI:** 10.1038/s41419-019-1425-4

**Published:** 2019-02-18

**Authors:** Tingting Yu, Xin Zhang, Haosong Shi, Jinge Tian, Lingling Sun, Xueming Hu, Wenqiang Cui, Dongping Du

**Affiliations:** 1 Pain Management Center, Shanghai Jiao Tong University Affiliated Shanghai Sixth People’s Hospital, Shanghai, 200233 China; 2 Center for Translational Pain Medicine, Department of Anesthesiology, Duke University School of Medicine, Durham, NC USA; 3 Department of Otorhinolaryngology, Shanghai Jiao Tong University Affiliated Sixth People’s Hospital, Shanghai, 200233 China; 4 Department of Anesthesiology, The First Affiliated Hospital of Zhengzhou University, Zhengzhou, 450052 China; 5 Department of Integrative Medicine and Neurobiology, State Key Laboratory of Medical Neurobiology, School of Basic Medical Science, Institutes of Brain Science, Collaborative Innovation Center for Brain Science, Institute of Acupuncture and Moxibustion, Fudan Institutes of Integrative Medicine, Fudan University, Shanghai, 200032 China

## Abstract

Peripheral nerve injury causes neuropathic pain and microglia activation. P2Y12 receptors on microglia are thought to be a key player in the surveillance of the local environment, but whether or how these receptors are engaged in the cross-talk between microglia and neurons of the dorsal horn remain ambiguous. Using a rodent model of nerve injury-induced pain, we investigated the roles of P2Y12 in microglia activation, excitatory synaptic transmission, and nociceptive allodynia. We found that spinal nerve ligation (SNL) significantly increased the level of P2Y12 receptors specifically in the microglia of the ipsilateral dorsal horn. Injections of P2Y12 antagonists (MRS2395 or clopidogrel) attenuated microglia activation and increased the paw withdrawal latency in response to thermal stimuli on the ipsilateral side without affecting the basal threshold on the contralateral side. These effects on pain behaviors were replicated in P2Y12 knockout mice. Patch-clamp recordings further revealed that partial sciatic nerve ligation (PSNL)-induced excessive miniature excitatory postsynaptic currents (mEPSCs) were significantly attenuated in P2Y12 knockout mice. Moreover, we found that SNL activates the GTP-RhoA/ROCK2 signaling pathway and elevates the level of phosphorylated p38 mitogen-activated protein kinase (MAPK), which was inhibited by the P2Y12 antagonist. The phosphorylation of p38 MAPK was inhibited by a ROCK inhibitor, but not vice versa, suggesting that p38 MAPK is downstream of ROCK activation. Our findings suggest that nerve injury engages the P2Y12 receptor-dependent GTP-RhoA/ROCK2 signaling pathway to upregulate excitatory synaptic transmission in the dorsal horn. This cross-talk ultimately participates in the manifestation of nociceptive allodynia, implicating P2Y12 receptor as a potential target for alleviating neuropathic pain.

## Introduction

Nerve injury-induced neuropathic pain involves painful responses evoked by normally innocuous tactile stimuli, and it is one of the most challenging clinical problems^1^. However, the currently available therapeutics for this pathological pain are relatively limited.

Microglia play an important role in the process of pathological pain. As potent stimulators of microglia, extracellular nucleotides caught our attention^2^. They play roles in various functions by activating purinergic receptors expressed in microglia^3^. In the pathological course of nerve injury, ATP can be released or leaked from a variety of sources, such as primary afferent terminals, dorsal horn neurons, and spinal astrocytes^4^. The release or leakage of ATP after nerve injury can then activate the neighboring microglia.

Increasing evidence has emphasized the importance of P2 receptors for spinal microglia. These receptors, such as P2X4^5^ and P2X7^6^, have important roles in chronic pain. Among them, P2Y12, a P2Y metabotropic G-protein-coupled purinergic receptors, has become a new focus^7^. Research shows that P2Y12 is constitutively involved in cancer pain^8^, synaptic plasticity in the mouse visual cortex^9^ and ATP-induced membrane ruffling and chemotaxis^10,11^. P2Y12 is restrictively expressed on microglia in the central nervous system^12^. Once microglia are activated, neurotransmitters and inflammatory cytokines are released, which regulate neuronal function^13^, but whether P2Y12 is involved in the changes in neuronal function has never been reported before.

Neuropathic pain is thought to be initiated by a series of changes in the sensory processing system, such as the functional reorganization of sensory transmission or aberrant development of neural plasticity. Our focus is on the superficial dorsal horn, especially the substantia gelatinosa (SG) area, which is highly involved in modulating nociceptive transmission^14^. In a previous study, whole-cell patch-clamp techniques were adapted to SG neurons in a spinal cord slice with an attached dorsal root to investigate synaptic responses to peripheral nerve stimulation^15^. However, our method involved stimulating the SG neurons directly and then assessing the miniature excitatory postsynaptic current (mEPSC) changes. Furthermore, the shape of EPSCs is determined by many factors, such as the amount of presynaptically released glutamate, the properties of postsynaptic glutamate receptors and the time course of glutamate clearance from the synaptic cleft^16^.

Antagonists of P2Y12 have been reported to attenuate inflammatory and neuropathic pain^17,18^. In our study, we confirmed that P2Y12 is involved in the pathological activation of microglia, a process that is presumably involved in synapse remodeling and neural plasticity. We also confirmed the underlying molecular signaling pathway between P2Y12 and neuropathic pain, involving p38 mitogen-activated protein kinase (MAPK) and GTP-RhoA/Rho-associated coiled-coil-forming protein serine/threonine kinase 2 (ROCK2). Our data demonstrate that P2Y12 antagonists can potently inhibit the activation of microglia and the classic signaling pathway of microglia. Most important of all, P2Y12 knockout mice showed lower mEPSC increases after nerve injury than wild-type (WT) mice.

## Results

### Spinal nerve ligation increased the expression of P2Y12

**Fig. 1:**
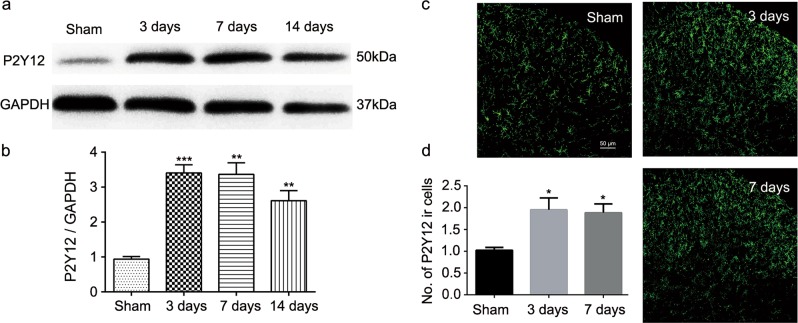
Spinal nerve ligation (SNL) increased the expression of P2Y12 in the spinal dorsal horn of rats. **a** Representative immunoblots of P2Y12 protein expression. Total cell lysates from the ipsilateral spinal cord were used, and GAPDH was used as an internal control. **b** Densitometric analysis (six rats per group) of P2Y12 proteins normalized to the loading control. **c** Representative immunofluorescence images of P2Y12 (green) staining in the spinal dorsal horn after SNL surgery. **d** Quantitative analysis of the number of P2Y12-immunoreactive cells per 400-μm visual field length per section per rat in the spinal dorsal horn after SNL surgery. Values are normalized to the sham group. *n* = 3 rats per group, with three sections per rat. Values are represented as mean ± SEM. *p < 0.05 vs. sham group, **p < 0.01 vs. sham group, ***p < 0.001 vs. sham group

The western blot analyses were used to determine the effect of spinal nerve ligation (SNL) surgery on P2Y12 expression in the spinal cord (Fig. 1a). Analysis of different time points revealed that the P2Y12 expression was increased from day 3 to 14 after SNL surgery compared to the expression in the sham group (Fig. 1a, b). Similarly, the fluorescence immunohistochemistry results showed that there was more P2Y12-immunoreactive cells per 400-μm length visual field per section on day 3 and 7 in the SNL group than in the sham group (Fig. 1c, d).

### MRS2395 and clopidogrel inhibited the development of tactile allodynia and thermal hyperalgesia induced by SNL surgery

**Fig. 2:**
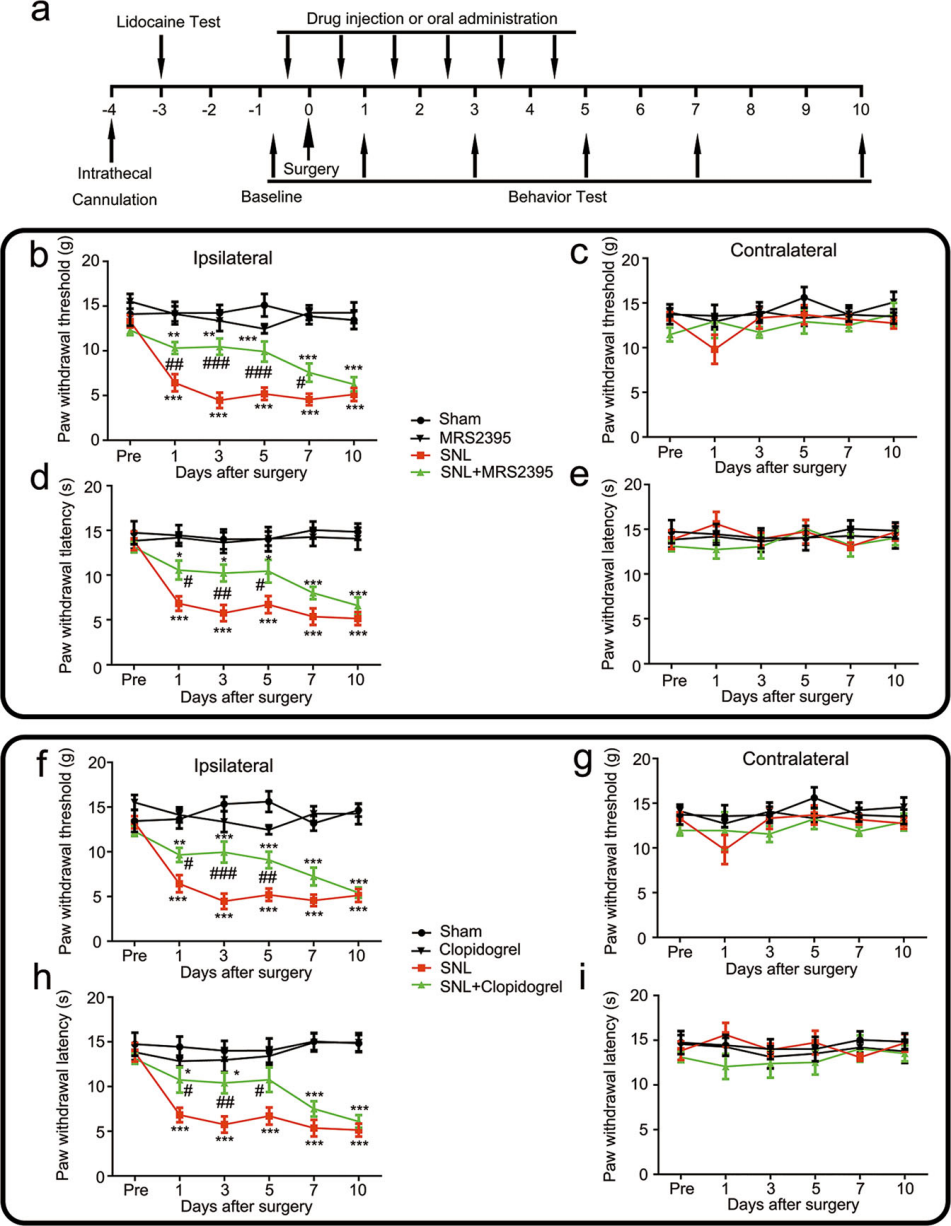
P2Y12 antagonists, MRS2395 and clopidogrel, attenuated spinal nerve ligation (SNL)-induced pain behavior in rats. **a** Schematic of the experimental timeline. Daily treatment with MRS2395 inhibited the mechanical allodynia (**b**) and thermal hyperalgesia (**d**) on the ipsilateral sides (**b**, **d**) and made no difference on the contralateral sides (**c**, **e**) after SNL surgery. MRS2395 was intrathecally injected three times a day, 200 μg per injection per rat, beginning from 1 day before surgery, and continuing for a further 5 days. MRS2395 reversed the mechanical allodynia for up to 7 days after surgery. Injury-induced thermal hyperalgesia was also suppressed by MRS2395 from day 1 to 5. There was no effect of MRS2395 on the mechanical allodynia and thermal hyperalgesia in the contralateral hind paw. Likewise, treatment with oral clopidogrel alleviated the mechanical allodynia (**f**) and thermal hyperalgesia (**h**) on the ipsilateral side (**f**, **h**), while the contralateral side was not affected (**g**, **i**). Oral clopidogrel was administered three times a day, 10 mg/kg per administration, beginning from 1 day before surgery and continuing for 6 days. Clopidogrel reversed the mechanical allodynia for up to 7 days after surgery. Injury-induced thermal hyperalgesia was also suppressed by clopidogrel from day 1 to 5. There was no effect of clopidogrel on the mechanical allodynia and thermal hyperalgesia in the contralateral hind paw. Values are represented as mean ± SEM, *n* = 6 rats per group. ^*^p < 0.05, vs. sham group, ^**^p < 0.01 vs. sham group, ^***^p < 0.001 vs. sham group; ^#^*p* < 0.05, vs. SNL group, ^##^*p* < 0.01, vs. SNL group, ^###^*p* < 0.001, vs. SNL group

The experimental design and timeline for the experimental rats are shown in Fig. 2a. SNL surgery decreased the threshold of tactile allodynia (based on 50% paw withdrawal threshold) (Fig. 2b–g) and thermal hyperalgesia (based on paw withdrawal latency) (Fig. 2d–i). Regarding mechanical nociceptive testing in the ipsilateral paw (Fig. 2b–f), the mechanical nociceptive threshold decreased from day 1 to 10 after SNL surgery, while after MRS2395 treatment, the threshold was partially reversed at days 1, 3, 5, and 7 after surgery (Fig. 2b). Simultaneously, oral clopidogrel administration attenuated the mechanical threshold at days 1, 3, and 5 after surgery in the ipsilateral paw (Fig. 2f). Thus, daily administration of MRS2395 or clopidogrel partially prevented the SNL-induced mechanical allodynia.

Regarding thermal latency testing (Fig. 2d–h), SNL surgery caused thermal hyperalgesia from day 1 to 10 in the ipsilateral paw compared with that in the sham group and it was alleviated by daily administration of MRS2395 (Fig. 2d) or clopidogrel (Fig. 2h) from day 1 to 5 after surgery. At the same time, the nociceptive behavior of the contralateral paw was also verified, and there were no significant differences in the mechanical withdrawal threshold (Fig. 2c–g) or thermal paw latency (Fig. 2e–i) in the SNL-only group compared with the MRS2395- or clopidogrel-treated group.

### P2Y12 was co-localized with ionized Ca^2+^-binding adapter molecule 1 (iba-1) in the spinal cord

**Fig. 3:**
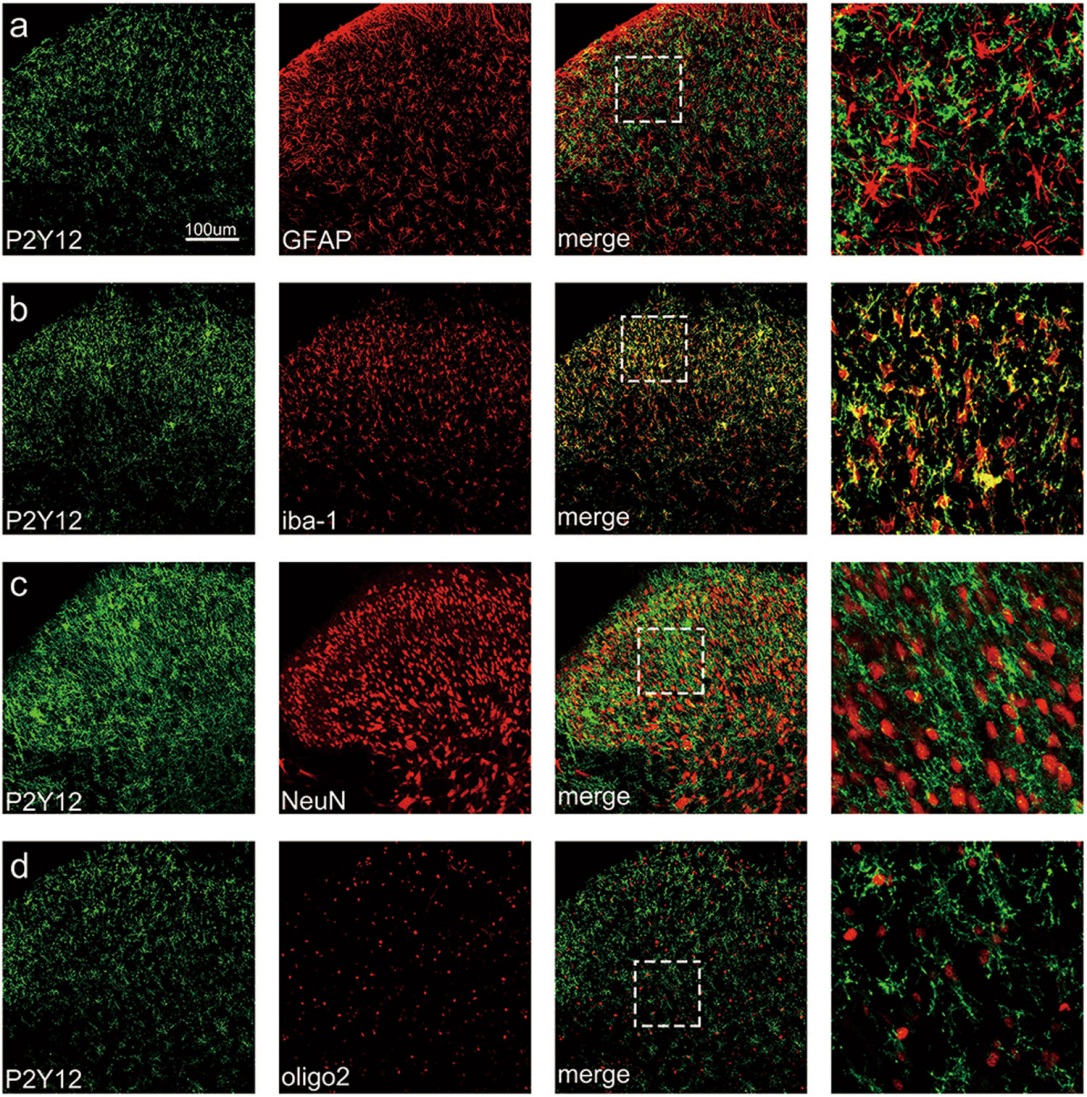
P2Y12 was co-localized with iba-1 in the spinal cord. Representative double-staining immunofluorescence images of P2Y12 (green) with GFAP (**a**, red), iba-1 (**b**, red), NeuN (**c**, red), and oligo 2 (**d**, red) in the spinal dorsal horn of rats 7 days after spinal nerve ligation (SNL). More than 30 cells per treatment group were included. Scale bars: 100 μm

As shown in Fig. 3, P2Y12 was strongly co-localized with iba-1 (a microglia activation marker) in the spinal cord 7 days after SNL surgery. Additionally, the higher-magnification images excluded the co-localization of P2Y12 with astrocytes, oligodendrocytes, or neurons based on double immunostaining involving P2Y12 plus glial fibrillary acidic protein (GFAP), oligo 2 or NeuN, respectively.

### P2Y12 antagonist changed the activation state of microglia in SNL rats

**Fig. 4:**
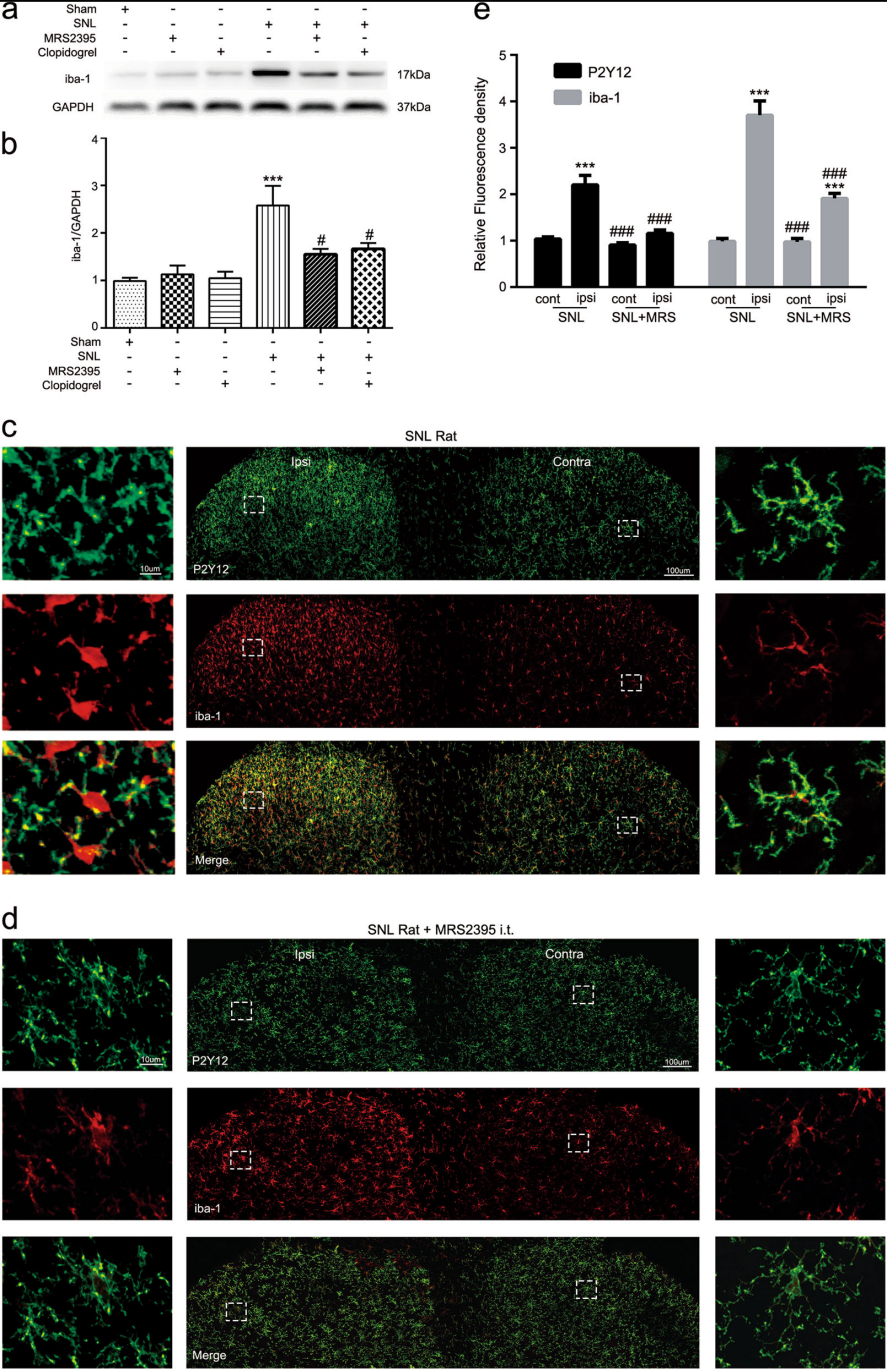
P2Y12 antagonist attenuated the spinal nerve ligation (SNL)-induced activation of microglia in the spinal dorsal horn of rats. **a** Representative immunoblots of iba-1 from total cell lysates of ipsilateral spinal cord tissue harvested 7 days after SNL surgery. GAPDH was used as the loading control. **b** Densitometric analysis (mean ± SEM, *n* = 6 rats per group) of iba-1 normalized to the GAPDH loading control. ^***^p < 0.001 vs. sham group; ^#^*p* < 0.05 vs. SNL group. **c** Representative confocal images at low and high magnification of both dorsal horns at postoperative day 7 following SNL in rats. Immunoreactivity of P2Y12 and iba-1 are shown in green and red, repectively. Scale bar: 100 and 10 μm for lower- and higher-magnification images, respectively. **d** Representative confocal images of MRS2395-treated rats after SNL at low and high magnification. High-magnification images showed that after injection of MRS2395, microglia morphology on the ipsilateral side transformed from an activated state (with swollen cell bodies and retracted processes) to a nonactivated state (with small cell bodies and more ramified processes). **e** Quantified immunoreactivity of P2Y12 and iba-1 per 400-μm visual field length per section per rat after SNL surgery was higher in the ipsilateral than the contralateral dorsal horn in SNL rats, but decreased in the dorsal horn of MRS2395-treated rats. *n* = 3 rats per group, with three sections per rat. Values are represented as mean ± SEM, ^***^p < 0.001 vs. contralateral side of each section, ^###^*p* < 0.001 vs. ipsilateral side of SNL group

To determine whether P2Y12 is involved in microglia activation, we used western blotting to analyze the expression of iba-1 in the ipsilateral spinal cord of rats in different treatment groups (Sham, SNL, SNL + MRS2395, SNL + clopidogrel) (Fig. 4a, b). MRS2395 or clopidogrel partially reversed the upregulation of iba-1 after SNL surgery (Fig. 4b), indicating that P2Y12 antagonists can inhibit the activation of microglia after nerve injury. Additionally, we used fluorescence immunohistochemistry to compare the fluorescence intensity of iba-1 in the ipsilateral or contralateral spinal dorsal horn in the different treatment groups. When SNL rats were intrathecally (i.t.) treated with MRS2397, the fluorescence intensity of iba-1 in the ipsilateral side was significantly decreased (Fig. 4c–e). Moreover, the morphology of microglia indicated a nonactivated state with small cell bodies and more ramified processes on the contralateral side in the SNL group and on both sides in the MRS2395 group (Fig. 4c, d). However, microglia were significantly activated on the ipsilateral side in the SNL group, with swollen cell bodies and retracted processes (Fig. 4c). Furthermore, the relative fluorescence density of iba-1 and P2Y12 in the ipsilateral spinal dorsal horn from the SNL group was significantly decreased after MRS2395 treatment (Fig. 4e). It is noteworthy that microglia proliferation occurred after nerve injury (Fig. S1). Lastly, the proportional increase in P2Y12 intensity was higher than the proportional increase in the number of P2Y12-positive cells (Fig. 1d), which indicates a simultaneous increase in the intensity of the cells.

### P2Y12 antagonist reduced the expression of p-p38 MAPK, and p38 MAPK inhibitor alleviated SNL-induced pain behavior

**Fig. 5:**
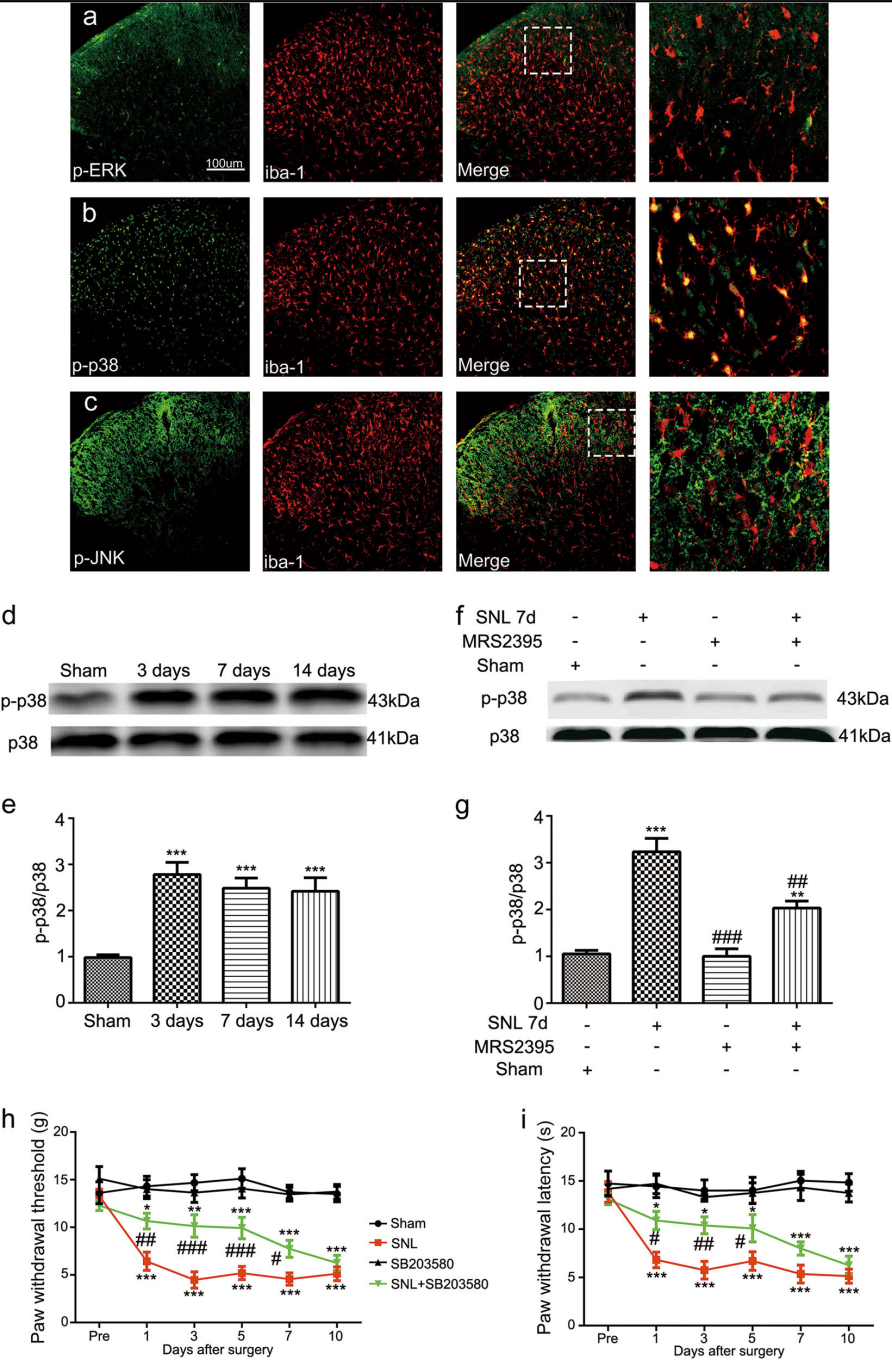
Activation of p38 MAPK occurred in spinal nerve ligation (SNL) rats and was attenuated by the P2Y12 inhibitor. Double immunofluorescence staining images of iba-1 (red) with p-ERK (**a**, green), p-p38 (**b**, green), and p-JNK (**c**, green). iba-1 and p-p38 were co-localized in the spinal cord harvested at day 7 after surgery. Scale bars: 100 μm. **d**, **f** Immunoblots of phosphorylated p38 from total cell lysates of ipsilateral spinal cord tissue harvested after surgery. Total p38 was used as an internal control. **e**, **g** Densitometric analysis of p-p38. **d** Phosphorylated p38 time dependently increased at days 3, 7, and 14 after surgery. **f** The level of p-p38 MAPK was increased after SNL surgery and MRS2395 (a P2Y12 inhibitor) partially suppressed the upregulation of p-p38 expression. **h**, **i** SB203580 (a p38 inhibitor) inhibited the mechanical allodynia (**h**) and thermal hyperalgesia (**i**) on the ipsilateral side after SNL surgery. SB203580 was intrathecally injected three times a day, 1 μg per injection per rat, beginning at 1 day before surgery and continuing for 6 days. SB203580 reversed the mechanical allodynia for up to 7 days after surgery. Injury-induced thermal hyperalgesia was also suppressed by SB203580 from day 1 to 5. SB203580 alone had no effect on the mechanical allodynia and thermal hyperalgesia. The data are presented as the mean ± SEM. *n* = 6 rats per group. ^*^p < 0.05, vs. sham group, ^**^p < 0.01, vs. sham group, ^***^p < 0.001, vs. sham group; ^#^*p* < 0.05, vs. SNL group, ^##^*p* < 0.01, vs. SNL group, ^###^*p* < 0.001, vs. SNL group

To determine the contributors to the increased P2Y12 expression in microglia, we assessed the possible downstream regulator: MAPK. We used immunofluorescence double staining to verify whether p-extracellular signal-regulated kinase (ERK), p-c-Jun N-terminal kinase (JNK), and/or p-p38 are activated in SNL rats and thus which MAPK signaling pathways are associated with microglia activation. As shown in Fig. 5a–c, p-p38 was highly expressed in the dorsal horn after SNL and exclusively co-localized with iba-1 (Fig. 5b), but this was not the case for p-ERK (Fig. 5a) or p-JNK (Fig. 5c). In addition, we found that the phosphorylation of p38 MAPK had the same time pattern as P2Y12 expression after SNL (Fig. 5d). Western blot analysis revealed that SNL increased the phosphorylation of p38 MAPK at days 3, 7, and 14 after surgery (Fig. 5d, e), and MRS2395 partially reversed the upregulation of p-p38 expression (Fig. 5f, g). Furthermore, as shown in Fig. 5h, i, an inhibitor of p38 MAPK, SB203580, partially suppressed pain behaviors in SNL rats. Mechanical allodynia was alleviated at days 1, 3, 5, and 7 after surgery (Fig. 5h). Thermal hyperalgesia was alleviated at days 1, 3, and 5 (Fig. 5i). SB203580 alone had no effect on the mechanical allodynia threshold or thermal hyperalgesia latency.

### Inhibition of RhoA/ROCK suppresses SNL-induced pain reflex and p38 phosphorylation after surgery

**Fig. 6:**
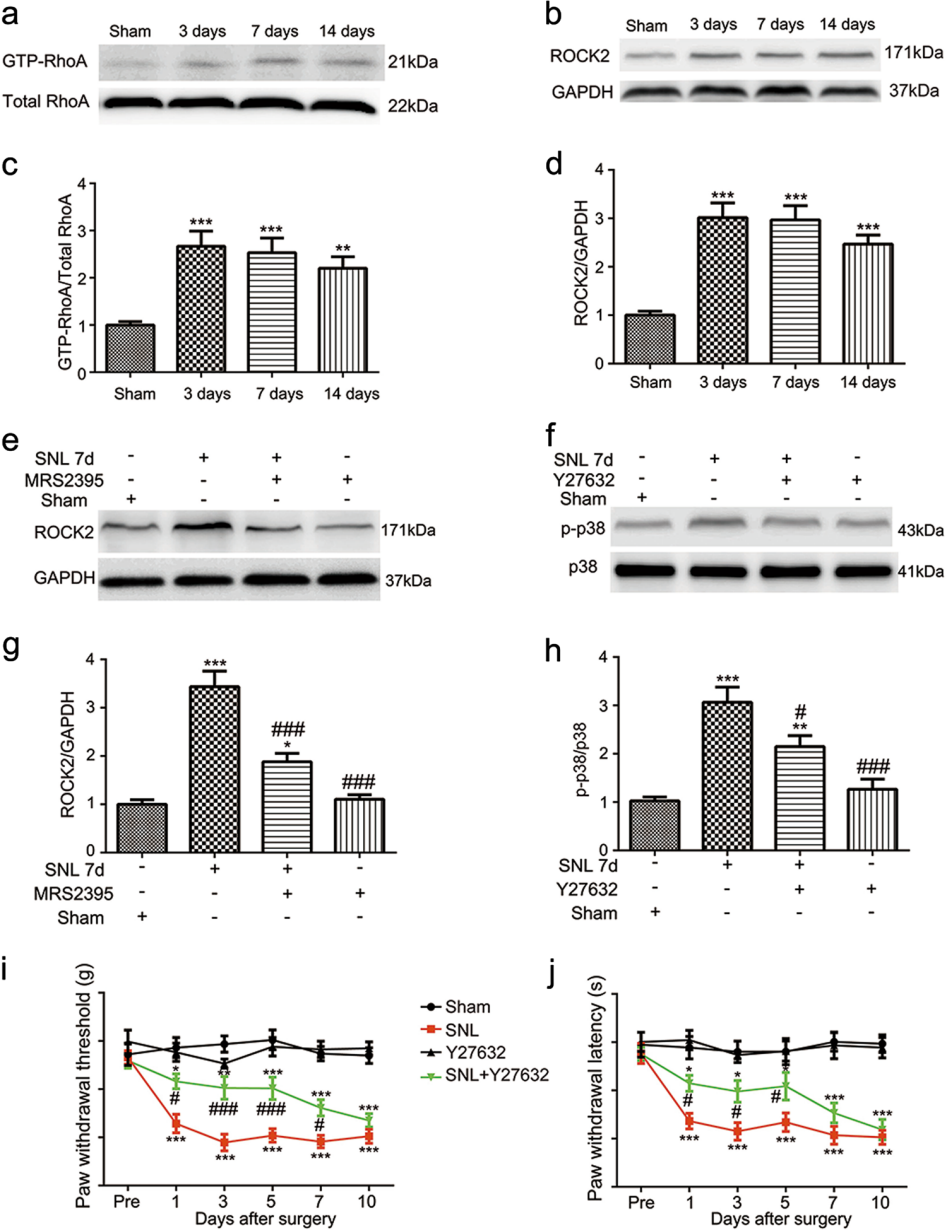
RhoA/ROCK is responsible for the spinal nerve ligation (SNL)-induced phosphorylation of p38 MAPK and hyperalgesia. Immunoblots of GTP-RhoA (**a**) and ROCK2 (**b**) from total cell lysates of ipsilateral spinal cord tissue. Densitometric analysis of GTP-RhoA (**c**) and ROCK2 (**d**) were normalized to the respective loading controls. The expression of GTP-RhoA and ROCK2 increased after SNL at days 3, 7, and 14 after surgery. Immunoblots of ROCK2 (**e**) and p-p38 (**f**) from total cell lysates of ipsilateral spinal cord tissue 7 days after SNL surgery. Densitometric analysis of ROCK2 (**g**) and p-p38 (**h**) were normalized to the respective loading controls. **g** The P2Y12 antagonist, MRS2395 partially suppressed the increased ROCK2 expression after nerve ligation. **h** The ROCK inhibitor, Y27632, reversed the increased phosphorylation of p38 MAPK induced by SNL. **i**, **j** Y27632 inhibited the mechanical allodynia (**i**) and thermal hyperalgesia (**j**) on the ipsilateral side after SNL surgery. Y27632 was intrathecally injected three times a day, 3 μg per injection per rat, beginning at 1 day before surgery and continuing for 6 days. Y27632 reversed the mechanical allodynia for up to 7 days after surgery. Injury-induced thermal hyperalgesia was also suppressed by Y27632 from day 1 to 5. Y27632 alone had no effect on the mechanical allodynia and thermal hyperalgesia. The data are presented as the mean ± SEM. *n* = 6 rats per group. ^*^p < 0.05, vs. sham group, ^**^p < 0.01, vs. sham group, ^***^p < 0.001, vs. sham group; ^#^*p* < 0.05, vs. SNL group, ^###^*p* < 0.001, vs. SNL group

Besides p38 MAPK, we also assessed another downstream regulator (RhoA/ROCK) of P2Y12 after spinal nerve injury. Western blotting was used to evaluate the expression of active-RhoA (GTP-RhoA, Fig. 6a) and ROCK2 (Fig. 6b) in the ipsilateral spinal cord after nerve injury. Their protein expression was time dependently increased at day 3, 7, and 14 after SNL surgery. We also evaluated the effect of Y27632 (ROCK inhibitor) on the expression of p-p38. Interestingly, Y27632 decreased the expression of p-p38 at day 7 after surgery (Fig. 6f–h), but the p38 inhibitor (SB203580) had no effect on the expression of ROCK2 (data not shown), which means that RhoA/ROCK2 signaling is upstream of p38 MAPK. However, the P2Y12 antagonist reversed the upregulation of ROCK2 after nerve injury (Fig. 6e–g), which means that P2Y12 is upstream of RhoA/ROCK2 signaling. Furthermore, Y27632 markedly attenuated the mechanical allodynia at days 1, 3, 5, and 7 (Fig. 6i) and thermal hyperalgesia at days 1, 3, and 5 after surgery (Fig. 6j) in the ipsilateral hind paw.

### P2Y12 knockout mice exhibited fewer nociceptive behaviors and lower mEPSCs in the ipsilateral superficial dorsal horn after nerve injury

**Fig. 7:**
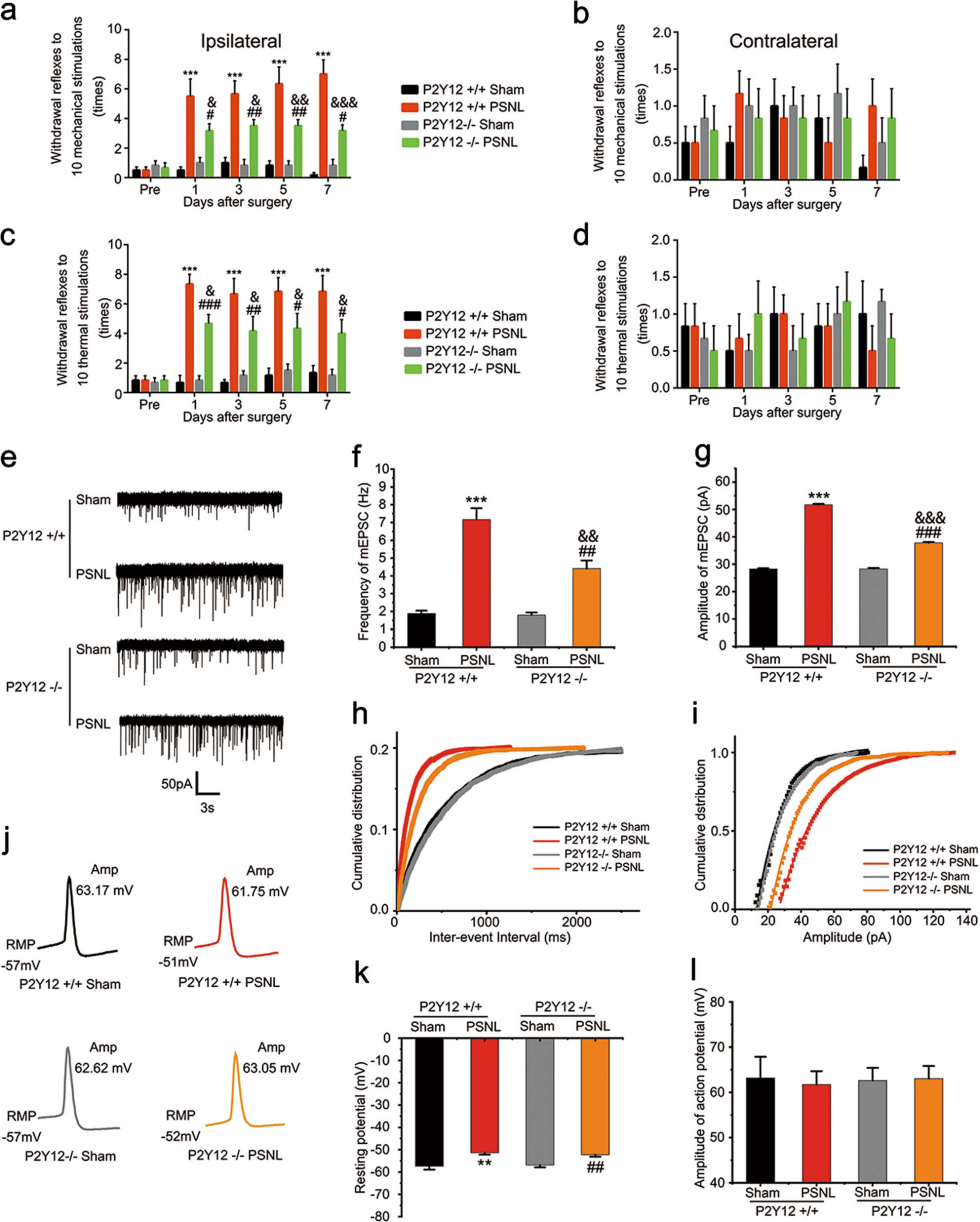
P2Y12^−/−^ mice exhibited alleviated nociceptive behavior and less excitatory synaptic transmission in lamina II neurons of spinal cord slice after partial sciatic nerve ligation (PSNL) surgery. Paw withdrawal reflexes in response to a defined mechanical stimulus (**a**, **b**) and thermal stimulus (**c**, **d**) were measured in wild-type and P2Y12^−/−^ mice before and after PSNL surgery in the ipsilateral (**a**, **c**) and contralateral (**b**, **d**) hind paw. Reduced mechanical allodynia and thermal hyperalgesia were recorded in the ipsilateral hind paw of P2Y12^−/−^ mice after PSNL surgery compared to wild-type mice (*n* = 6 mice per group). **e** Representative traces of miniature excitatory postsynaptic current (mEPSC) from lamina II (substantia gelatinosa [SG] area) neurons based on whole-cell recording. Statistics on mEPSC frequency (**f**) and amplitude (**g**) in different treatment groups (*n* = 10 neurons per group). Cumulative curves of mEPSC frequency (**h**, bin size = 25 ms) and amplitudes (**i**, bin size = 1 pA). There was no obvious difference between the two sham groups in mEPSC frequency or amplitude, while both the surgery groups exhibited higher frequencies and larger amplitudes, with lower values in the P2Y12^−/−^ group. **j** Representative traces of action potential recordings evoked by 1000 pA current injection lasting 2 ms from lamina II (SG area) neurons, and resting membrane potential was recorded using the *I* = 0 mode. Quantification of resting membrane potential (**k**) and action potential (**l**) (*n* = 10 neurons per group). ^**^p < 0.01 vs. P2Y12^+/+^ sham group, ^***^p < 0.001 vs. P2Y12^+/+^ sham group; ^#^*p* < 0.05 vs. P2Y12^−/−^ sham group, ^##^*p* < 0.01 vs. P2Y12^−/−^ sham group, ^###^*p* < 0.001 vs. P2Y12^−/−^ sham group; ^&^*p* < 0.05 vs. P2Y12^+/+^ PSNL group, ^&&^*p* < 0.01 vs. P2Y12^+/+^ PSNL group, ^&&&^*p* < 0.001 vs. P2Y12^+/+^ PSNL group

We used knockout mice to verify the effect of P2Y12 on neuronal properties. First, we confirmed that the mechanical and thermal withdrawal reflexes in the ipsilateral hind paw of P2Y12 knockout mice were also increased at days 1, 3, 5, and 7 after surgery compared with the sham mice (Fig. 7a–c). However, P2Y12 knockout mice showed fewer reflexes in response to the defined stimuli than the WT mice (Fig. 7a–c).

Previous studies have shown that P2Y12 is critical for chronic pain^8,17,18^, but how it affects pain is unclear. Thus, we wondered whether P2Y12 contributes to synaptic transmission and thereby influences neuropathic pain. As shown in our electrophysiological results, after SNL surgery, both mEPSCs frequency and amplitude in the spinal lamina II neurons were consistently increased in WT mice (frequency: WT Sham: 1.89 ± 0.16 Hz, WT PSNL: 7.17 ± 0.63 Hz, Fig. 7e–h; amplitude: WT Sham: 28.26 ± 0.27 pA, WT PSNL: 51.75 ± 0.30 pA, Fig. 7e–i; *n* = 10 in each group). As expected, besides the behavioral improvement compared to WT mice, P2Y12 knockout mice demonstrated less facilitation of mEPSCs after nerve injury. (Frequency: KO Sham: 1.80 ± 0.14 Hz, KO PSNL: 4.41 ± 0.46 Hz, Fig. 7e–h; amplitude: KO Sham: 28.30 ± 0.31 pA, KO PSNL: 37.82 ± 0.30 pA, Fig. 7e–i; *n* = 10 in each group). As the shape of EPSCs is determined by the amount of presynaptically released glutamate and the properties of postsynaptic glutamate receptors^16^, our results indicated that both presynaptic glutamate release and postsynaptic glutamate receptors may be enhanced in the spinal lamina II after SNL surgery, and this enhancement was reduced by P2Y12 knockout. As for resting membrane potential (RMP) and action potential (AP), postsurgery P2Y12 knockout mice did not show any significant differences compared to the postsurgery WT mice (RMP: WT Sham: −57.38 ± 1.53 mV, WT PSNL: −51.36 ± 0.82 mV, KO Sham: −56.88 ± 1.02 mV, KO PSNL: −52.24 ± 0.87 mV; AP: WT sham: 63.17 ± 4.70 mV, WT PSNL: 61.75 ± 2.92 mV, KO Sham: 62.62 ± 2.78 mV, KO PSNL: 63.05 ± 2.77 mV; *n* = 10 in each group (Fig. 7j–l)).

## Discussion

In the present study, we found that P2Y12 antagonists attenuated SNL-induced nociceptive thermal hyperalgesia, which concurs with previous research on mechanical allodynia^7^. Consistent with previous studies^18,19^, P2Y12 expression in the dorsal horn was highly restricted to microglia (Fig. 3). As shown in Fig. 1a, the expression of P2Y12 was increased in the ipsilateral spinal cord after SNL surgery. The main reason for the P2Y12 upregulation in the ipsilateral spinal cord could be the increased expression of P2Y12 in individual microglia, but another possible reason could be microglia proliferation after nerve injury (Fig. S1).

Notably, we also employed another P2Y12 antagonist, clopidogrel^20^, which is a well-known, effective, orally administered antithrombotic compound targeting P2Y12 in platelets with safety profiles from an extensive clinical program^21^. It has been reported that the effect of clopidogrel on P2Y12 is dependent on its active metabolite generated during hepatic metabolism, and transfer across the blood–brain barrier has also observed^22^. We observed a significant effect of 10 mg/kg oral clopidogrel, which alleviated tactile allodynia and thermal hyperalgesia (Fig. 2f–h) in the ipsilateral hind paw after SNL. A previous study^7^ reported that a higher dose (25 mg/kg) achieved a rather longer-lasting effect on existing tactile allodynia.

In all events, whether microglia activation is influenced by P2Y12 should be determined. We confirmed that SNL surgery increased the expression of iba-1 in the ipsilateral spinal cord of rats. However, MRS2395 and clopidogrel partially reversed the upregulation of iba-1, indicating that P2Y12 antagonists can inhibit the activation of microglia in SNL rats (Fig. 4a, b). We also compared the fluorescence intensity of iba-1 between the ipsilateral and contralateral spinal cord in different treatment groups at 7 days after surgery. The fluorescence intensity of iba-1 and P2Y12 was significantly decreased after treatment with MRS2395 (Fig. 4c, d) in SNL rats. The morphology of microglia in the contralateral spinal cord of SNL rats indicated a nonactivated state, with small cell bodies and more ramified processes (Fig. 4c). However, the microglia in the ipsilateral side was significantly activated, exhibiting swollen cell bodies and retracted processes (Fig. 4c). However, after treatment with MRS2395, the activated state of microglia in the ipsilateral spinal cord diminished and they became nonactivated state with ramified processes (Fig. 4d). This transformation indicates that the activation of microglia was obviously suppressed by MRS2395.

Peripheral nerve injury induces a dramatic activation of intracellular signaling cascades, such as MAPK signaling^23,24^, many researches have been conducted on p38 MAPK^25–27^. Consistent with these studies, our results verified that a robust increase of p38 MAPK phosphorylation occurred after SNL surgery (Fig. 5d, e). The behavioral experiments (Fig. 5h, i) showed that the inhibitor of p38 alleviated the pain behavior induced by SNL, which implies that the activation of p38 in spinal microglia is a critical step in the pathogenesis of neuropathic pain. Additionally, the P2Y12 antagonist effectively inhibited the expression of p-p38 (Fig. 5f, g). Pharmacological inhibition of P2Y12 decreased the phosphorylation of p38 MAPK, but not vice versa, as p38 inhibition does not affect P2Y12 mRNA during neuropathic pain^28^. This suggests that p38MAPK could be a downstream target of P2Y12 to modulate pain hypersensitivity.

RhoA, a member of a small molecular G-protein family, is involved in many cellular functions, including cytoskeletal rearrangement, cell motility, phagocytosis, and intracellular trafficking^29^. RhoA cycles between inactive (GDP-bound) and active (GTP-bound) forms, its active form interacts with downstream effectors to regulate cellular functions. The best matched downstream effector of RhoA is Rho kinase (ROCK). Activation of the RhoA/ROCK pathway has been observed in various central nervous system disorders, such as stroke and inflammation in the brain^30^. Importantly, RhoA/ROCK has been reported to play a key role in the activation of p38 MAPK in the spinal cord^1^. It has been reported that RhoA/ROCK induces over-activation of the cytoskeleton, which may act as a scaffold for the trafficking of nociceptive signaling factors. Moreover, the inhibition of RhoA/ROCK leads to the alleviation of neuropathic pain in mice^31–34^. Our results show that rats pretreated with a ROCK inhibitor exhibited less hyperalgesia after SNL (Fig. 6i, j). Therefore, the RhoA/ROCK pathway has a crucial role in neuropathic pain. Dynamic remodeling of the cytoskeleton, especially of actin filaments^35^, which provides a network for trafficking intracellular proteins may be trafficked to improve our research.

Most important of all, we confirmed that nerve injury triggered changes in excitatory synaptic transmission in the ipsilateral spinal cord of mice. After nerve injury, there was an obvious increase in the frequency and amplitude of mEPSCs in layer II neurons in WT mice. However, in global genetic P2Y12 knockout mice, both presynaptic and postsynaptic changes were reduced (Fig. 7e–i), consistent with the decrease in pain behavior in P2Y12 knockout mice (Fig. 7a–c). These results indicate that P2Y12 was involved in the enhanced excitatory synaptic responses in SG neurons after nerve injury. Thus, P2Y12 in the lamina II is an attractive candidate protein related to neural plasticity. To our knowledge, no modulation effect of P2Y12 on synaptic transmission has been reported before.

**Fig. 8:**
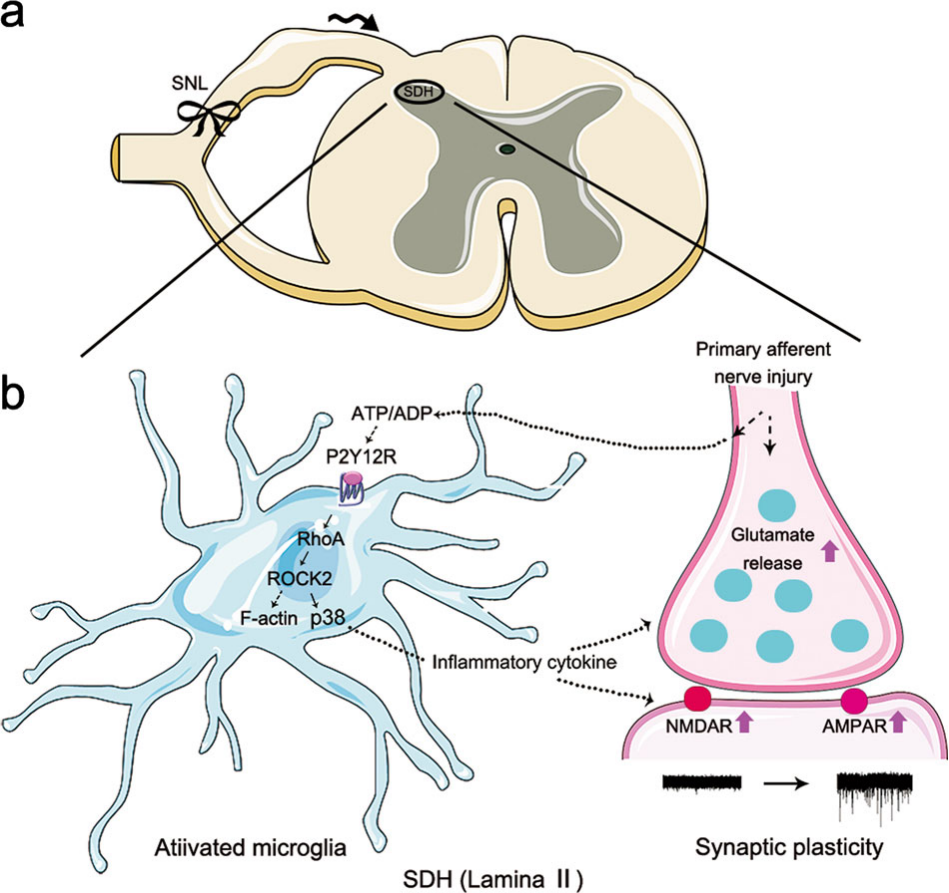
Schematic illustration of neuron–microglia interactions in the superficial dorsal horn (SDH) lamina II (substantia gelatinosa [SG] area) in neuropathic pain. Nerve ligation induced hyperexcitability of primary sensory neurons causes excessive release of ATP from central afferent terminals, which can activate adjacent microglia in the spinal dorsal horn. As a result, P2Y12 is upregulated in microglia and activated by ATP or metabolic products of ATP such as ADP. Upon activation, P2Y12 activates RhoA/ROCK signaling to induce F-actin stabilization and thereby induce microglia morphology changes. Meanwhile, microglia synthesize and release inflammatory cytokines via the phosphorylation of p38MAPK, leading to enhanced excitatory synaptic transmission and neuronal hyperactivity in the dorsal horn. After such neuromodulation processes in the spinal pain circuit, pain sensitivity is enhanced. Solid lines indicate the pathways and mechanisms demonstrated in this study. Dashed lines indicate possible pathways and mechanisms

In conclusion, our results suggest that P2Y12 is necessary for neuron–microglia interactions underlying neuropathic pain in the superficial dorsal horn lamina II (SG area) (Fig. 8). We provided evidence on P2Y12, which involved pain-related postsynaptic enhancement via microglia activation in the spinal lamina II after nerve injury. This study provides new insight into the effect of P2Y12 on pain circuitry.

## Materials and methods

### Animals

Adult male Sprague–Dawley rats weighing 250–300 g were obtained from Shanghai Laboratory Animal Center at the Chinese Academy of Science (Shanghai, China). Male C57BL/6J (WT) and P2Y12 knockout mice were gifts from Junling Liu’s lab (Department of Biochemistry and Molecular Cell Biology, School of Medicine, Shanghai Jiao Tong University, Shanghai, China). The rats were housed in groups of five in cages before surgery and individually after surgery. All the animals were housed on bedding in a room maintained at a constant temperature of 22–23 °C with an alternating 12/12 h light/dark cycle, and water and food were available ad libitum. Before every operation, six animals were randomly chosen for each group. The protocol was approved by the Animal Care and Use Committee of the Sixth People’s Hospital Affiliated to Shanghai Jiao Tong University (SYXK [Shanghai, China] 2011-0128, January 1, 2011). All studies involving animals are reported in accordance with the ARRIVE (Animals in Research: Reporting in vivo Experiments) guidelines. All efforts were made to minimize the suffering and reduce the number of animals used. The overall experimental design involving rats is illustrated in Fig. 2a.

### Spinal nerve ligation neuropathic pain model establishment in rats

To produce a spinal nerve ligation (SNL) model, the rats were anesthetized with isoflurane inhalation in 100% oxygen (induced at 5% and maintained at 2%) and placed in a prone position. A midline incision was made at the L4–S2 level. The left paraspinal muscles were separated from the spinous processes, and the L6 transverse process was removed to expose the L4 and L5 spinal nerves. The L5 spinal nerve was then isolated and tightly ligated with 4-0 silk thread^36^. A sham surgery involved the same procedure without the ligation of the L5 spinal nerve.

### Intrathecal cannulation

For repeated injection of drugs, i.t. cannulation was performed using a previously described method^37^. Briefly, the rats were anesthetized with isoflurane inhalation in 100% oxygen (induced at 5% and maintained at 2%), and then a 6-cm PE-10 catheter (Becton Dickinson, Sparks, MD, USA) was inserted into the subarachnoid space at the L4–L5 level. During the surgery, the concentration of isoflurane was increased when necessary. Involuntary movements of the tail or hind limb were regarded as signs of dura penetration. The catheter was then advanced 1.5 cm into the subarachnoid space to reach the site of the lumber enlargement. The external end of the catheter was sealed using heat. The correct location of the catheter was tested by i.t. injection of lidocaine (2%, 10 μl) on the next day, which reversibly paralyzed the bilateral hind limb for 10–15 min. Before drug administration, the rats were allowed to recover for 3 days. To avoid infection, penicillin was intraperitoneally (i.p.) administered during the surgery.

### Drugs administration

Before delivering the drugs, the catheterized rats were briefly anesthetized with isoflurane and placed in a transparent Plexiglas box. They were then slowly injected with drugs (1 μl/min) via the exteriorized portion of the catheter with a micro-syringe (Hamilton, Reno, NV, USA) containing 10 μl of the drug followed by flushing with 10 μl normal saline (Baxter Healthcare, New York, NY, USA).

Different groups of rats (*n* = 6 per group) were treated with the P2Y12 antagonist MRS2395 (200 μg in 10 μl 5% DMSO, Sigma, St. Louis, MO, USA); the p38 MAPK inhibitor SB203580 (1 μg in 10 μl 10% DMSO, Sigma, St. Louis, MO, USA); the ROCK inhibitor Y27632 (3 μg in 10 μl normal saline, Sigma, St. Louis, MO, USA), or normal saline (10 μl) or 10% DMSO (10 μl) as controls. The oral drug administrated rats were gavaged with the orally active P2Y12 antagonist clopidogrel (10 mg/kg, Abcam, Cambridge, MA, USA). The drugs were delivered three times per day for 6 days, from 1 day before SNL surgery to 5 days after surgery.

### Tests of paw withdrawal threshold and latency

To evaluate the behavioral response to mechanical stimulation, the 50% paw withdrawal threshold was determined using the up–down method as previously described^38^. Briefly, the rats were placed in a transparent plastic cage with a wire mesh bottom. After 30 min of acclimatization, a series of von Frey filaments (0.4, 0.6, 1.0, 1.4, 2.0, 4.0, 6.0, 8.0, 10.0, and 15.0 g; Stoelting, Wood Dale, IL, USA) were sequentially applied to the plantar surface of the ipsilateral and contralateral paws. Brisk withdrawal and hind paw-licking were recognized as positive responses. If continuous positive or negative responses occurred until the exhaustion of the stimulus set, values of 0.4 and 15 g were assigned, respectively. Changes in general behavior including repetitive movements, vocalization or activity level were noted throughout testing.

Thermal hyperalgesia was measured using an IITC Plantar Analgesia Meter (IITC Life Science Inc., Woodland Hills, CA, USA) to measure paw withdrawal latency as described previously^39^. Briefly, each animal was placed in a box containing a smooth, temperature-controlled glass floor. The heat source was focused on a portion of the hind paw, which was flush against the glass, and a radiant thermal stimulus was delivered to that site. The stimulus was shut off when the hind paw moved, or after 20 s to prevent tissue damage. The time from the onset of radiant heat to the endpoint was the paw withdrawal latency. The radiant heat intensity was adjusted to obtain a basal paw withdrawal latency of 10–12 s in control rodents. Thermal stimuli were delivered three times to each hind paw at 5–6 min intervals. All of the behavioral tests were assessed by an examiner who was blind to the treatment groups.

### Western blotting

Rats were sacrificed at days 0, 3, 7, and 14 after surgery, and the spinal cord was collected for western blot analysis. Under deep anesthesia with pentobarbital (60 mg/kg, i.p.), rats were subjected to a rapid intra-cardiac infusion of ice-cold saline containing heparin. The lumbar enlargements were then harvested and immediately stored at −80 °C in a refrigerator. The dorsal portions of the lumber enlargements (100 mg) were homogenized in 400 μl ice-cold RIPA lysis buffer (Beyotime, Shanghai, China) containing a cocktail of protease and phosphatase inhibitors. After incubation on ice for 15 min, the lysate was centrifuged at 12000×*g* and 4 °C for 20 min to isolate the proteins. The protein concentrations of the samples were determined with a BCA protein assay kit (Beyotime, Shanghai, China). Samples containing 50 μg protein were denatured by heating at 100 °C for 5 min, separated on sodium dedecyl sulfate-polyacrylamide gels, and transferred onto polyvinylidene difluoride membranes (Millipore, Billerica, MA, USA). The membranes were blocked with 5% nonfat milk in Tris-buffered saline with Tween (TBST) for 2 h at room temperature. They were then incubated overnight at 4 °C with primary antibodies: rabbit anti-P2Y12 (1:1000, Anaspec Inc., MA, USA), rabbit anti-GAPDH (1:5000, Hangzhou HuaAn Biotechnology Co., Ltd., Hangzhou, China), mouse anti-iba-1 (1:500, Merck & Co Inc., NJ, USA), mouse anti-β-tubulin (1:5000, Hangzhou HuaAn Biotechnology Co., Ltd, Hangzhou, China), rabbit anti-pp38 (1:1000, CST, Danvers, MA, USA), rabbit anti-p38 (1:1000, Abcam, Cambridge, MA, USA), mouse anti-RhoA (1:500, Abcam, Cambridge, MA, USA), and rabbit anti-ROCK2 (1:500, Abcam, Cambridge, MA, USA). All primary antibodies were diluted in 5% nonfat milk in TBST. The membranes were then washed with TBST and incubated for 2 h at room temperature with corresponding horseradish peroxidase-conjugated secondary antibodies. Enhanced chemiluminescence reagent (Thermo Fisher Scientific, Rockford, IL, USA) were used to detect the signal, and ImageQuant Ai600 (General Electric Co., Kenilworth, NJ, USA) was used to visualize the image. The results were analyzed and quantified by ImageJ software (version 2.0, NIH, Bethesda, MA, USA). Each western blot analysis was performed at least six times, and consistent results were obtained.

### RhoA activity assay

According to the manufacturer’s directions, active GTP-bound RhoA was detected in the lysates from spinal dorsal horn tissue in rats. The lysates were subjected to a pull-down assay using a RhoA activation assay kit (Abcam, Cambridge, MA, USA). Briefly, lysates were incubated for 1 h with anti-active-RhoA mouse monoclonal antibody and Protein A/G Agarose Bead slurry at 4 °C on a rotator. Bead-precipitated proteins were fractionated and then immunoblotted with antibody against RhoA.

### Fluorescence immunohistochemistry

Regarding the fluorescence immunohistochemistry, rats were anesthetized and transcardially perfused with 4% cold paraformaldehyde on day 7 after surgery, lumbar spinal cords were harvested, postfixed for 2 h at 4 °C in 4% paraformaldehyde, and then dehydrated sequentially in 10%, 20 and 30% sucrose overnight for 3 days. The spinal sections were transversely cut into 30 μm slices in a cryostat and then washed in TBST. The sections were first blocked with 0.3% Triton X-100 in 5% donkey serum for 1 h at 20–25 °C. They were then incubated overnight at 4 °C with the following primary antibodies: goat anti-iba-1 (1:400, Abcam, Cambridge, MA, USA), mouse anti-GFAP (1:400, 1:1000, CST, Danvers, MA, USA), mouse anti-NeuN (1:400, Millipore, Billerica, MA, USA), mouse anti-oligo2 (1:200, Millipore, Billerica, MA, USA), rabbit anti-P2Y12 (1:1000, Anaspec Inc., MA, USA), rabbit anti-Ki67 (1:1000, Abcam, Cambridge, MA, USA), rabbit anti-pp38 (1:1000, CST, Danvers, MA, USA), rabbit anti-p38 (1:1000, Abcam, Cambridge, MA, USA), rabbit anti-pJNK (1:500, Abcam, Cambridge, MA, USA), and rabbit anti-pERK (1:2000, Abcam, Cambridge, MA, USA). The sections were then washed three times for 15 min in TBST and incubated for 1 h at about 20–25 °C with corresponding secondary antibodies (conjugated to Alexa Fluor 488 or 594; 1:2000; Invitrogen, Carlsbad, CA, USA). The immunofluorescence images were captured with a confocal scanning laser microscope (Fluo View FV1000, Olympus Co., Tokyo, Japan).

### Partial sciatic nerve ligation neuropathic pain model establishment in mice

Male C57BL/6J (WT) mice (P10-P14) and P2Y12 knockout mice (P10–P14) were anesthetized with sodium pentobarbital (40 mg/kg, i.p.) and received partial sciatic nerve ligation (PSNL) surgery^40^. Briefly, after deep anesthesia, a tight ligature was placed around the dorsal half of the left sciatic nerve using 8-0 nylon such that one-half to one-third of the sciatic nerve was ligated. In the sham group, the skin and muscle were just incised to expose the nerve. The wounds were then closed, and the pups were immediately transferred to a jacketed water-filled heating pad maintained at around 37 °C. Each pup was covered with the padding from the cage where they lived with their mother to cover all surgery-related odors. Upon full recovery from anesthesia, usually about 2 h after surgery, the pups were returned to their mothers. At several time points (0, 1, 3, 5, and 7 days) after the PSNL surgery, nociceptive behavior was tested. After every behavior test, each pup moved around in the padding from the cage where they lived with their mother for at least 1 h to hide odor changes from their mother.

### Tests of frequency of withdrawal reflexes

Regarding the nociceptive behavior tests in mice, we counted the number of withdrawal reflexes in response to a sequential series of ten stimulations^14^ to the plantar surface of the ipsilateral hind paw (with the injured nerve that underwent PSNL) and contralateral hind paw using a 1 g von Frey filament and 2.5 s radiant thermal stimulus (the radiant heat intensity was adjusted to obtain a basal paw withdrawal latency of 10–12 s in WT control mice). As the mice rarely responded to the mechanical and thermal stimuli prior to surgery, the elevated behavioral responses evident after surgery were defined as allodynia. The mice were acclimatized to the environment and investigator for at least 1 h before the tests. Each stimulus was applied to the middle area between the footpads on the plantar surface of the hind paw and maintained for approximately 2 s. A withdrawal response was considered valid only if the hind paw was removed completely from the platform. If a mouse walked immediately after stimulation instead of lifting the paw, the test was reapplied. A trial consisted of six tests at 5 min intervals. After the last behavior test at 7 days after surgery, the mice were prepared for the electrophysiology test.

### Preparation of spinal cord slices

Spinal cord slices obtained from mice (P17–P21) at 7 days after surgery were used for electrophysiological assessments. We conducted the procedure according to a protocol described in detail previously^41^ with some modifications. Briefly, the mice were deeply anesthetized with sodium pentobarbital (60 mg/kg, i.p.) and then subjected to a rapid intra-cardiac infusion of ice-cold saline containing heparin. The lumber enlargements were quickly removed and immersed in ice-cold oxygenated high-sucrose artificial cerebrospinal fluid (ACSF) containing (in mM): NaCl, 95; KCl, 1.8; KH2PO_4_, 1.2; CaCl_2_, 0.7; MgCl_2_, 1; NaHCO_3_, 26; sucrose, 50; and glucose, 15. The pH was adjusted to pH 7.4 (osmolality 300–310 mOsm), and the fluid was oxygenated with 95% O_2_ and 5% CO_2_. To identify the ipsilateral side, a sharp knife was used to make a deep mark in the contralateral side. The spinal cord was then transversely sliced into 300 μm sections. In preparation for the electrophysiology recordings, the slices were incubated in ACSF containing (in mM) NaCl, 124; KCl, 5; KH_2_PO_4_, 1.2; CaCl_2_, 0.5; MgSO_4_, 3; NaHCO_3_, 24; and glucose, 10. The pH was adjusted to 7.4 (osmolality 300–310 mOsm), and the fluid was oxygenated with 95% O_2_ and 5% CO_2_ for 30 min at 37 °C.

### Electrophysiology

The lamina II (SG area) in the superficial dorsal horn was visually identified as reported before^42^ in spinal slices with a 60× water immersion objective attached to an upright microscope (ECLOPSE FN1, Nikon, Tokyo, Japan). mEPSCs were recorded from lamina II neurons using the whole-cell patch-clamp technique with an amplifier (EPC-10 usb, HEKA, Lambrecht, Germany), using the voltage-clamp mode with a pipette potential of −70 mV. Patch electrodes were pulled from borosilicate capillary glass using a vertical pipette puller (PC-10, Narishige, Tokyo, Japan) and had a resistance of 4–7 MΩ. The pipettes were filled with an intracellular solution containing (in mM): 97.5 K-gluconate, 32.5 KCl, 0.5 ethylene glycol tetraacetic acid (EGTA), 40 4-(2-hydroxyethyl)-1-piperazineethanesulfonic acid (HEPES) and 1 MgCl_2_ (adjusted to pH 7.2 with Tris base). When recording mEPSCs, the extracellular solution contained ACSF with 10 μM bicuculline and 1 μM strychnine to block GABAergic and glycinergic synaptic currents, and 100 nM tetrodotoxin (TTX) to block APs. When the electrode tip touched the cell membrane, gentle suction was applied to form a tight seal (serial resistance > 2 GΩ). At −70 mV command voltage, additional suction was applied to rupture the cell membranes. After obtaining recordings using the whole-cell mode, the recording was switched to current-clamping mode and the RMP was recorded. To compare the relative comprehensive excitability of the spinal lamina II neurons in different groups, we also examined the AP evoked by a 2 ms intracellular depolarizing current of 1000 pA. The electrode capacitance and liquid junction potential were compensated and data were filtered at 1–3 kHz and sampled at 3–10 kHz using a Dell computer equipped with Clampex software (Molecular Devices, Sunnyvale, CA, USA). The access resistance, which was monitored throughout the experiment, 15–30 MΩ. All electrophysiological recordings and data analyses were conducted by researchers blind to the treatment group.

### Statistical analysis

GraphPad Prism 5 (GraphPad Software Inc., San Diego, CA, USA) was used to conduct the statistical analyses. Changes in detected protein expression were tested using one-way repeated-measures analysis of variance (ANOVA), followed by the Dunnett’s multiple comparison test. Changes in behavior responses to von Frey filaments and radiant heat stimuli over time among the groups were tested using two-way repeated-measures ANOVA, followed by the Bonferroni post hoc test. The electrophysiology data were acquired by Clampfit 10.2 software (Molecular Devices, Sunnyvale, CA, USA) and analyzed using SPSS 22.0 software (IBM Corp, Armonk, NY, USA). The relative expression of target proteins in different groups was normalized to GAPDH, and the phosphorylation level of target proteins was compared with their total level. We set the mean value of the sham group to 1, and standardized every set of data accordingly. All data analyses were performed by researchers who were blind to the treatment groups. All data are presented as the mean ± SEM. Statistical differences were considered significant if *p* < 0.05.

## Supplementary information

Figure S1

Supplemental figure legends
