# Supplementary figures and images for: P2Y12 regulates microglia activation and excitatory synaptic transmission in spinal lamina II neurons during neuropathic pain in rodents
Source: Cell Death Dis. 2019 Feb 18;10(3):165. doi: 10.1038/s41419-019-1425-4 (PMC6379416; doi:10.1038/s41419-019-1425-4)

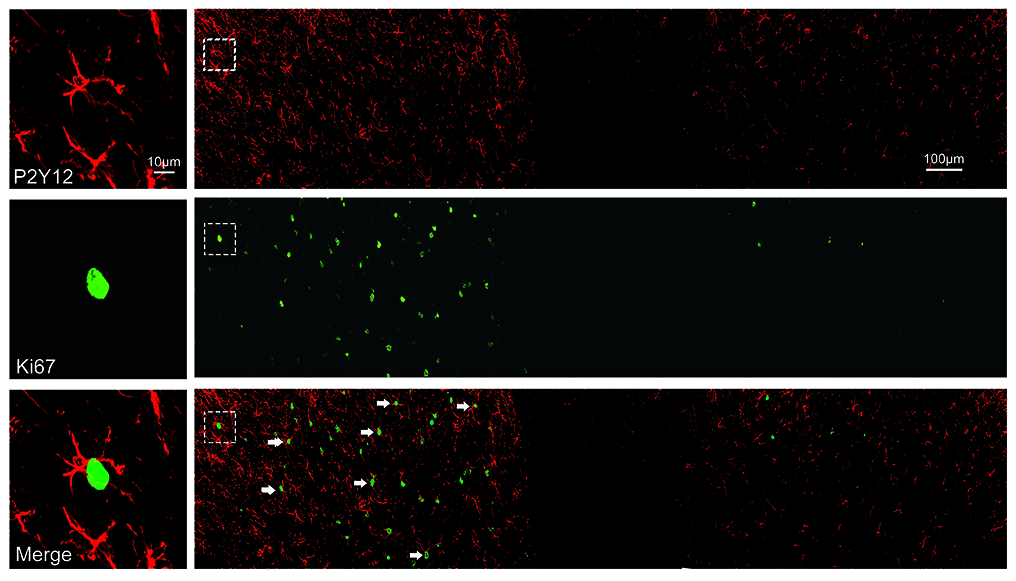

Supplement: Supplementary file 1 — Figure S1 [file 41419_2019_1425_MOESM1_ESM.tif]
