# Supplementary material for: P2Y12 regulates microglia activation and excitatory synaptic transmission in spinal lamina II neurons during neuropathic pain in rodents
Source: Cell Death Dis. 2019 Feb 18;10(3):165. doi: 10.1038/s41419-019-1425-4 (PMC6379416; doi:10.1038/s41419-019-1425-4)
Supplement: Supplementary file 2 — Supplemental figure legends [file 41419_2019_1425_MOESM2_ESM.docx]

Fig.S1 **Microglia proliferation occurs in the ipsilateral dorsal horn after nerve injury.**

Immunostaining for Ki67 (a nuclear protein expressed in all phases of the cell cycle except the resting phase) was performed to gain a snapshot of dividing microglia after spinal nerve ligation surgery at day 14. Robust spinal microglia proliferation occurred on the ipsilateral side (the arrow shows the co-localization of P2Y12 and Ki67), whereas the contralateral side only had a few signals. Scale bar: 100 μm and 10 μm for lower- and higher-maginification images, respectively.
